# The Symptomatic Outcomes of Cholecystectomy for Gallstones

**DOI:** 10.3390/jcm12051897

**Published:** 2023-02-28

**Authors:** Daniel Mønsted Shabanzadeh

**Affiliations:** Department of Surgery, Nordsjaellands Hospital Hilleroed, Dyrehavevej 29, DK-3400 Hilleroed, Denmark; daniel.moensted.shabanzadeh@regionh.dk

**Keywords:** cholelithiasis, cholecystolithiasis, laparoscopic cholecystectomy, postcholecystectomy syndrome, review

## Abstract

Cholecystectomy is the definite treatment for symptomatic gallstones, and rates are rapidly rising. Symptomatic complicated gallstones are generally treated with cholecystectomy, but there is no consensus on the clinical selection of patients with symptomatic uncomplicated gallstones for cholecystectomy. The aim of this review is to describe symptomatic outcomes before versus after cholecystectomy in patients with symptomatic gallstones as reported in prospective clinical studies and to discuss patient selection for cholecystectomy. Following cholecystectomy, resolution of biliary pain is high and reported for 66–100%. Dyspepsia has an intermediate resolution of 41–91% and may co-exist with biliary pain but may also develop following cholecystectomy with an increase of 150%. Diarrhea has a high increase and debuts in 14–17%. Persisting symptoms are mainly determined by preoperative dyspepsia, functional disorders, atypical pain locations, longer duration of symptoms, and poor psychological or physical health. Patient satisfaction following cholecystectomy is high and may reflect symptom alleviation or a change in symptoms. Comparison of symptomatic outcomes in available prospective clinical studies is limited by variations in preoperative symptoms, clinical presentations, and clinical management of post-cholecystectomy symptoms. When selecting patients with biliary pain only in a randomized controlled trial, 30–40% still have persisting pain. Strategies for the selection of patients with symptomatic uncomplicated gallstones based on symptoms alone are exhausted. For the development of a selection strategy, future studies should explore the impact of objective determinants for symptomatic gallstones on pain relief following cholecystectomy.

## 1. Introduction

Cholecystectomy is currently the only definite treatment for symptomatic gallstones. Cholecystectomy rates have been rapidly rising in European countries within the past decades [1,2] and possibly also in pediatric populations [3]. Treatment of gallstones and cholecystectomy generally cause high healthcare costs [4]. High proportions of persistent symptoms and pain following cholecystectomy have previously been reported when reviewing prospective clinical studies [5], indicating impaired clinical guidelines and practice. This review provides an update on the symptomatic outcomes of cholecystectomy for symptomatic gallstones and discusses the selection of patients for surgery.

Gallstones are highly prevalent in general populations of the US and Europe and depend largely on ethnicity, age, and female sex [6]. The natural course of gallstones has been explored in one cohort, which included people with ultrasound screen-detected gallstones and who remained uninformed of their gallstone status. Only 18% developed symptomatic gallstones requiring hospital admission during long-term follow-up, and about half were due to symptomatic uncomplicated gallstones. The conclusion was that the natural course of gallstones is less aggressive than previously anticipated and that the vast majority of prevalent gallstones remain clinically silent and require no further treatment [7]. The current clinical challenge is selecting the right patients with symptomatic uncomplicated gallstones who will benefit the most from cholecystectomy.

The symptomatic complicated gallstones include acute cholecystitis, common bile duct stones, cholangitis, and pancreatitis while the uncomplicated include symptomatic gallstones in the absence of complications [8]. Cholecystectomy is recommended for all complicated gallstones [9,10,11,12,13,14]. In presence of symptomatic uncomplicated gallstones, current clinical practice guidelines have no consensus on which symptoms or patient characteristics should guide selection for cholecystectomy [9,10,11,12,14,15]. A randomized controlled trial of observation versus cholecystectomy concluded that the non-operative strategy caused fewer symptomatic complicated gallstones during follow-up when compared to post-cholecystectomy complications. Although the study proved the feasibility of observation in the presence of symptomatic uncomplicated gallstones [16], long-term follow-up of the population also showed cholecystectomy to be the preferred treatment [17]. Most practice guidelines recommend cholecystectomy for symptomatic uncomplicated gallstones [9,10,11,12,14], while observation is either disregarded [9] or only suggested [13]. The pooled postoperative morbidity following cholecystectomy is reported to be 1.6–5.3%, including bile duct injuries in 0.32–0.52% [18].

Etiologies for post-cholecystectomy symptoms have recently been systematically reviewed and pooled into (1) co-existent diseases such as gastroesophageal reflux, peptic ulcer, or functional disorders; (2) physiological changes including duodenogastric reflux causing gastritis or bile acid malabsorption causing diarrhea; (3) retained or newly formed gallstones; (4) sphincter of Oddi dysfunction; (5) psychological distress; or (6) surgical complications such as common bile duct injury or incisional hernia. Symptoms may debut, but most often they persist [19]. Common bile duct stones may present with symptoms within 6 days to 18 years following laparoscopic cholecystectomy [20]. Gallstones may also be retained or newly formed in remnants of the cystic duct following cholecystectomy or of the gallbladder following a subtotal cholecystectomy. Estimates for complications due to retained or newly formed gallstones following cholecystectomy are reported with wide ranges between studies [19]. Although there are many possible reasons for post-cholecystectomy symptoms, a third of patients have no identified causes [21].

Several study limitations may bias estimates of symptomatic outcomes following cholecystectomy in published clinical studies. The risk of bias may be due to either uncontrolled estimates without preoperative symptom assessment [22,23,24,25,26,27] or to retrospective assessments following surgery with a risk of recall bias [28,29]. On the other hand, estimates from prospective clinical studies may be limited by the short duration of follow-up [30,31,32,33]. Both retrospective and prospective studies may have a risk of attrition bias with either high or unreported proportions of patients lost to follow-up [34,35,36] or otherwise incomplete postoperative assessments such as unanswered questionnaires [37].

This review describes symptomatic outcomes before versus after cholecystectomy in patients with symptomatic gallstones. The focus is on declines or inclines in the proportion of patients with symptoms, on the debut of symptoms, and on the determinants of post-cholecystectomy symptoms as reported in prospective clinical studies.

## 2. Biliary Pain Definitions and Determinants

Symptoms of biliary pain ascribed to gallstones have traditionally been termed “biliary colic” by pioneers in medical practice such as Sir William Osler. The original symptom complex was defined by a sudden debut, of intense and agonizing pain, localized in the right hypochondrium or epigastrium, with projection to the shoulder, and a duration of hours to days or possibly even weeks [38]. Similar but more simple definitions of biliary pain were later accepted in a series of gallstone screening studies in Italy during the 1980–90′ies which included abdominal pain during the last five years, with a duration of more than 30 min, and localized in the right hypochondrium and/or epigastrium [39,40,41]. Most definitions of biliary pain share similarities in regard to pain localization and duration, but may also include nausea or vomiting [42,43], not being relieved by bowel movements [42,44], a possible association to food ingestion [42], and forcing one to stop all current activity, to lie down, or to take analgesics [45,46]. All these biliary pain definitions rely on expert opinions or consensus statements rather than empirical evidence (Table 1).

In studies exploring gallstone prevalence in general or clinical populations, it has generally been challenging to associate gallstones with a specific symptom or complex. Although more or less strict definitions of biliary colic have been associated with gallstone prevalence when compared to controls without gallstones, the diagnostic accuracy and predictive test values are very low [47].

Only a few determinants for the development of symptomatic gallstones have been identified when studying gallstone cohorts through long-term follow-up. In the cohort including persons uninformed and unaware of the presence of gallstones at baseline as described above, determinants for the development of symptomatic uncomplicated gallstones were pain localized in the epigastrium, with a duration of hours, of moderate to extreme intensity, and with the need for analgesics. Pain at night determined symptomatic complicated gallstones only [48]. These findings share similarities with the originally described “biliary colic”, except for pain projection. Pain projection has only been associated with gallstones in patients with emergency admissions in a cross-sectional study and with incident gallstones identified through multiple ultrasound examinations in a population-based cohort study [49,50].

Determinants for the development of symptomatic gallstones of a more objective character have also been identified in cohort studies. These include younger age, female sex, multiple gallstones [7,51], gallstone size of 1 cm or above [7,51,52], higher body mass index (BMI) [51,53], gallbladder polyps, tobacco smoking, and a number of comorbidities [51].

The mechanisms involved in subjective symptom generation are not fully understood but are believed to include a gallstone passage to the duodenum or an obstruction of the gallbladder or bile ducts causing biliary distention [38,54]. This should then activate visceral sensory neurons and create a sensation of pain [55]. In support of these long-held traditional beliefs, experimental distention of the gallbladder has been shown to cause pain in the right hypochondrium or epigastrium in patients with acute cholecystitis [56].

**Table 1:** Definitions of biliary pain in different types of studies.

Consensus-Based Definitions	
Gallstone screening studies	
GREPCO—Rome Group for the Epidemiology and Prevention of Cholelithiasis (1984) [39]	Abdominal pain in the last five years Duration of 30 min or more Localized in right hypochondrium and/or epigastrium
Chianciano population study (1994) [40]	
MICOL—Multicenter Italian Study on Cholelithiasis (1995) [41]	
Sirmione Study (1987) [44]	Same definition as [39,40,41]
	+ not relieved by bowel movements
Clinical studies	
Ros and Zambon (1987) [42]	Steady pain in the right hypochondrium/epigastrium
	Nausea or vomiting
	Duration of at least one hour
	Associated or not with food ingestion
	Unrelated to bowel movements
	Unassociated with discomfort at urination
Heaton et al. (1991) [45]	Abdominal pain attacks during the last year
	Duration of 30 min or more
	Localized in the upper abdomen
	Forces one to stop activities, lie down, or take analgesics
Martinez de Pancorbo et al. (1997) [46]	Abdominal pain in the right hypochondrium and/or epigastrium
	Forces one to lie down or take analgesics
Mertens et al. (2010) [43]	One or more of upper abdominal pain, nausea, and vomiting
Clinical practice guidelines	
NICE—National Institute for Health and Care Excellence (2014) [9]	No recommendation due to lack of research
UMHS—University of Michigan Health System (2014) [14]	Severe episodic pain
	Localized in right upper abdominal quadrant/epigastrium
	May be nocturnal
	Occasionally postprandial
	Possibly tenderness
EASL—European Association for the Study of the Liver (2016) [10]	Episodic attacks of severe pain
	Localized in right upper abdominal quadrant/epigastrium
	Radiation to the right back or shoulder
	Duration of at least 15–30 min
	A positive response to analgesics
Association of Upper Gastrointestinal Surgeons of Great Britain and Ireland, Royal College of Surgeons (2016) [15]	Pain localized in right upper quadrant/epigastrium
	Frequently radiating to the back
	Duration of several minutes to hours
	Often occurring at night
Dutch Society for Surgery (2017) [11]	Biliary colic
	Radiating pain clockwise to the back
	A positive response to analgesics
German Society for Digestive and Metabolic Diseases and German Society for Surgery of the Alimentary Tract (2018) [12]	Pain attacks localized in right upper quadrant/epigastrium
	Duration of more than 15 min
	Possible projection to back and right shoulder
	Possible nausea and vomiting
Data-based Definitions in Prospective Studies	
Prospective cohort studies	
Shabanzadeh et al. (2017) [48]	Pain localized in the epigastrium
	Moderate to extreme intensity
	Duration of hours
	Need for analgesics

## 3. Limitations of Studies Exploring Symptomatic Outcome

Most prospective clinical studies exploring symptomatic outcomes of cholecystectomy report results from unselected patient populations including both symptomatic complicated and uncomplicated gallstones. The latter may include symptoms of unspecified pain or other abdominal complaints, such as dyspepsia. Most of these studies of unselected populations report the presence of either biliary or unspecified pain at baseline [42,43,57,58,59,60,61,62,63,64,65,66,67,68,69,70,71,72,73,74,75,76,77,78,79], and only a few include patients with mostly dyspepsia [58]. Exploration of selected study populations with dyspepsia as the main complaint at baseline has been performed in only a few studies [80,81,82], and one study has performed a subgroup analysis of patients with dyspepsia [43].

The identification of underlying diseases for post-cholecystectomy symptoms requires diagnostic examinations [19]. Most available studies do not report the performance of examinations at clinical follow-up or the identification of causes for post-cholecystectomy symptoms. Few studies report the performance of post-cholecystectomy cholangiography, endoscopy, ultrasound, or blood samples [42,57,59,60,62,63,65,66,69,71,83,84]. Even fewer studies report treatments for identified underlying diseases causing post-cholecystectomy symptoms before the final clinical assessments or how they managed patients in the analysis of the symptomatic outcome for cholecystectomy [42,57,66,83,84].

## 4. Pooled Symptoms Outcome

Most of the studies reporting pooled symptom outcomes include populations with pain at baseline [42,43,60,61,62,63,64,65,66,68,69,70,74,77,78,83,85,86,87]. Resolution of the symptom indicating cholecystectomy is reported for 44–92% [42,43,60,61,62,63,64,65,66,68,69,70,74,77,78,81,83,85,86,87,88,89,90,91,92,93]. Persisting symptoms are reported for 8–56% [42,43,60,62,63,64,65,66,68,70,74,77,78,81,83,85,86,87,88,89,90,91,92,93] and a debut of symptoms in 1–34% of patients [43,61,68,74,75,83]. The wide ranges of symptom resolution, persistence, and debut are either due to the heterogeneous selection of patients for surgery in the available study populations or to changes caused by surgery. Due to the variation in clinical presentations and baseline symptoms in study populations, the interpretation of pooled symptom outcome estimates following cholecystectomy should be cautious. On the other hand, patient satisfaction following cholecystectomy is high, with reported proportions of 66–97% [61,62,64,65,70,73,75,86,87,92,93,94]. This may indicate that, although symptoms may not be resolved completely, they may be significantly improved in most patients.

Symptom resolution is determined by a preoperative history of cholecystitis [70] and a higher age [62]. Persisting symptoms are determined by preoperative dyspeptic symptoms [63,70], symptom duration over 6 months, gastritis [93], poor self-rated health [70], higher Gastrointestinal Symptom Rating Scale, higher Hopkins Symptom Checklist for assessment of anxiety and depression [81], higher trait anxiety [43], higher Psychological Symptom Score [93], and an American Society of Anesthesiologists (ASA) score of III-IV [70]. Unsatisfying or unsuccessful cholecystectomy outcome is determined by flatulence, duration of days or more for pain episodes [59], symptom duration over 6 months, and poor self-rated health [70]. Both younger age and an age above 55 years have been associated with persisting symptoms [59,62] (Table 2). Female sex has been associated with postoperative symptoms in a retrospective study only [24].

## 5. Pain Outcome

Outcomes for biliary colic or otherwise defined biliary pain are the most frequently reported following cholecystectomy, with a resolution of 66–100% [42,43,57,59,60,61,62,63,64,65,66,67,68,73,74,76,78,79,97]. Biliary pain does not seem to debut in available studies [62], and no studies report an incline following cholecystectomy (Table 3).

Undefined or atypical pain is estimated with a wide range of changes following cholecystectomy, from a resolution of 41–99% [43,62,65,69,70,72,75,76,77] to an incline of 22–41% [71,74], and to a debut in 0–7% [62,69,75,84] (Table 3). Complaints of pain that cannot be defined as biliary pain may have multiple causes that co-exist but otherwise are unrelated to gallstones [19,79]. These outcome estimates should, therefore, also be interpreted with caution.

Identified determinants for pain resolution are no heartburn, lower visual analogue scale, pain attacks [78], pain awakening at night [77], pain frequency of one per month or less [77], symptom duration of one year or less [77,87], no use of pain medication, no previous abdominal surgery [78], higher Gastrointestinal Quality of Life Index [87], and higher age [77,78]. Postoperative persistence of pain is determined by dyspepsia [68], functional dyspepsia and/or irritable bowel syndrome [79], atypical pain locations or lower abdominal pain [74,77], flatulence, food intolerance [74], abnormal bowel pattern (diarrhea or constipation), often feeling bloated [77], pain attacks every month [74], and psychic vulnerability [83] (Table 2). Female sex has been associated with postoperative pain in retrospective studies only [25,26,27].

## 6. Dyspepsia Outcome

Dyspepsia is reported with an intermediate resolution of 41–91% [42,43,57,58,62,75,95,98] and to debut in 0–6% [58,62,75,84] following cholecystectomy in studies including unselected patient populations as described above.

Definitions of dyspepsia have been without consensus and changed much throughout time. Flatulent dyspepsia was once a multi-symptomatic syndrome thought to be caused by gallstones but was also found to be due to other disorders [99]. The more recently described functional dyspepsia is defined according to the Rome IV criteria as one or more of bothersome postprandial fullness, early satiation, epigastric pain, or burning in the absence of structural disease at endoscopy [100]. These varying and broad definitions of dyspepsia make it challenging to assess changing symptoms between studies.

It may be of more value to assess the single symptoms commonly thought to comprise the dyspepsia complex. Heartburn is reported to resolve for 43–72% of patients [57,69,70,75,76,80,93,95] and to debut in 1–8% [69,70,75]. Regurgitation or reflux resolves for 24–92% [60,62,69,74,76,80,93,95] and debuts in 2–14% [62,69,89]. Nausea resolves for 54–98% [59,60,61,65,66,69,70,72,75,76,93,97] and debuts in 0–2% [69,70,75]. Vomit resolves for 57–100% [59,65,66,69,70,74,75,76] and debuts in 0–1% [69,70,75]. Fat or food intolerance resolves for 61–100% [60,61,62,66,70,74,75,97] and debuts in 2–13% [60,62,70,75,84]. Indigestion or postprandial heaviness resolves for 41–93% [59,60,61,66,80,93]. Flatulence resolves for 29–79% [59,60,61,62,65,69,70,75,80] and debuts in 0–10% [62,69,70,75,84] (Table 3). Generally, dyspeptic symptoms have intermediate resolution in studies of unselected populations. Single studies have reported inclines for regurgitation or reflux of 37% [89], nausea of 100%, and vomiting of 243% [71]. These studies have very small-sized study populations, and the results have not been reproduced in larger studies.

In studies of populations where dyspepsia is the main complaint at baseline, a minor resolution of dyspepsia for 17–28% has been reported [80,82]; however, populations are small and highly selected. One study including a larger population undergoing elective laparoscopic cholecystectomy found a high incline of 150% in patients reporting dyspeptic symptoms without biliary symptoms following cholecystectomy (preoperative 14% versus postoperative 35%) (Table 3). In comparison, symptom relief in patients with preoperative biliary symptoms without or with dyspeptic symptoms was 88% and 77%, respectively [43]. Dyspeptic symptoms may thereby develop in unselected patient populations. Mechanisms of dyspeptic symptom development following cholecystectomy have been suggested in experimental studies and include duodenogastric reflux, histological gastritis [71,101,102,103], and impaired postprandial gastric emptying [104].

Determinants for postoperative dyspeptic symptoms are preoperative dyspepsia [43,68], non-specific symptoms, psychotropic medication [43], and a middle age range of 40–69 years when compared to the younger or older [95] (Table 2).

It seems that biliary pain and dyspeptic symptoms may co-exist, and that cholecystectomy resolves dyspeptic symptoms in unselected populations where most patients report pain at baseline. However, dyspepsia may also incline or debut following cholecystectomy. Resolution of dyspeptic symptoms in populations where they are the main preoperative complaint seems inconsistent based on the currently available studies. Larger studies exploring populations with functional dyspepsia or, alternatively, analysis of subgroups should be performed in the future. Until further, cholecystectomy is not indicated for the presence of dyspepsia only.

## 7. Diarrhea Outcome

Diarrhea seems to develop rather than resolve following cholecystectomy and is reported with high inclines of 40–850% [66,92,93,96,105,106] and to debut in 14–17% [84,92]. Available studies show a generally low preoperative prevalence of 2–22% [66,92,93,96,105,106] (Table 3). Determinants for postoperative diarrhea are younger age below 50 years [92,96] and higher BMI group [96] (Table 2).

Diarrhea is believed to be due to continuous bile excretion following cholecystectomy, which may also cause bile acid malabsorption [107]. Another proposed mechanism includes a shortened gut transit due to accelerated colonic passage [108]. More recently, a decreased microbial diversity in patients with post-cholecystectomy diarrhea has been demonstrated when compared to patients without diarrhea following cholecystectomy or to healthy controls [109,110].

Estimating the prevalence of post-cholecystectomy diarrhea is challenging. Bile acid malabsorption is diagnosed with a SeHCAT (selenium homocholic acid taurine) test that shows low absorption of radiolabelled bile acids. Tests are usually performed several years following cholecystectomy [111,112]. A retrospective study with a follow-up of four years has shown diarrhea to debut in 12% [108]. Prospective studies with multiple postoperative assessments report inclining proportions of patients with diarrhea within the first month following cholecystectomy, only to decline again during the following months [93,96,106,113]. Available prospective studies have a maximum follow-up duration of 12 months, which, therefore, may exaggerate the incidence of diarrhea [66,92,93,96,105,106]. A more exact prevalence of post-cholecystectomy diarrhea has yet to be explored in future clinical studies. Such studies should have longer and more systematic follow-ups of an entire cholecystectomy cohort including both symptom assessments and functional tests.

## 8. Other Functional Disorders Outcome

Constipation is reported with minor declines of 0–47% following cholecystectomy [66,74,93,96,105]. Mechanisms of changing bowel function following cholecystectomy, including increased colonic transit, have also been suggested to correct constipation [105,108]. However, changes in constipation following cholecystectomy are of a minor magnitude and may, until further notice, also be ascribed to random changes during follow-up.

Abdominal bloating or distention is reported to resolve for 21–73% of patients following cholecystectomy in unselected populations [59,61,65,66,74,76,97]. Bloating or distention is part of irritable bowel syndrome according to the Manning criteria [114]. It is also associated with irritable bowel syndrome and other functional bowel disorders according to the Rome IV criteria [115]. Currently, no studies have explored symptom outcomes following cholecystectomy in populations with mainly functional gastrointestinal disorders.

A recent large prospective observational study found that patients with functional dyspepsia and/or irritable bowel syndrome at baseline were significantly less likely to be pain-free six months after cholecystectomy when compared to those without (41% vs. 64%). Biliary colic was effectively resolved following cholecystectomy, independently of concomitant functional dyspepsia and/or irritable bowel syndrome [79]. Other studies have also found that patients with functional gastrointestinal disorders are less likely to resolve symptoms [85] and that patients with irritable bowel syndrome have fewer improvements in gastrointestinal quality of life scores following cholecystectomy [116]. Patients with irritable bowel syndrome have an increased risk of cholecystectomy that is not due to an increased risk of gallstones [117].

Currently, it seems that patients with mainly functional gastrointestinal disorders should be carefully selected for cholecystectomy based on the presence of biliary pain if offered surgery at all.

## 9. Selection of Patients for Cholecystectomy

The high rates of biliary pain resolution reported in prospective clinical studies indicate a true benefit of cholecystectomy, and it appears beneficial to select patients with biliary pain for cholecystectomy and, vice versa, refrain from surgery in its absence. A recent large randomized controlled trial, the SECURE trial, has challenged this assumption. Patients with uncomplicated gallstones and abdominal pain were selected for cholecystectomy through allocation to either a restrictive or a usual care strategy. In the usual care strategy, the selection was at the discretion of the physician. In the restrictive strategy, cholecystectomy was advised only to those patients who fulfilled a five-criteria symptom complex including (1) severe pain attacks, (2) pain duration of 15–30 min or longer, (3) pain localized in the epigastrium or right upper quadrant, (4) pain radiating to the back, and (5) a positive response to simple analgesics. If cholecystectomy was deferred accordingly at the first clinical assessment, the need was reconsidered during follow-up assessments. At one-year follow-up, only 72% versus 98% of patients allocated to the restrictive strategy were treated per protocol when compared to the usual care strategy. Thereby, a large cross-over of patients to cholecystectomy was seen in the restrictive strategy arm. The primary outcome was the proportion of patients being pain-free at follow-up, which was no different for the restrictive and usual care strategies (64% versus 63%, respectively). The only identified differences were fewer cholecystectomies in the restrictive strategy. The study concluded suboptimal pain reduction for both strategies [118]. Parallel with the SECURE trial, a prospective cohort study, the Success trial, developed a prediction model for clinically significant pain reduction following cholecystectomy, which was validated in the study population of the SECURE trial [78]. The identified determinants for pain reduction have been referred to in this present review (see Section 5 and Table 2).

The SECURE trial demonstrated the clinical challenges of withholding patients from a protocolled treatment and, even more, it challenged the definitions and predictive value of biliary pain. Despite protocolled attempts to select patients for cholecystectomy based on strict biliary pain definitions, the proportions of patients with persisting pain were unacceptably high.

Patient selection for cholecystectomy relying only on patient-reported symptoms has been clinical practice for a long time but is suboptimal if the aim is a pain-free state following surgery. Although prediction scores for symptomatic outcomes of cholecystectomy are of limited availability and even fewer are validated [78], available studies seem to have exhausted the exploration of preoperative patient-reported symptoms for post-cholecystectomy outcomes. Future clinical studies should focus on exploring more objective variables for symptom resolution following cholecystectomy. Determinants for the development of symptomatic gallstones as identified in previous larger cohort studies (see Section 2) are candidate explorative variables for future studies [7,51,52,53]. Due to a consensus on the need for cholecystectomy in the treatment of symptomatic complicated gallstones in clinical practice guidelines, future studies should confine to populations of symptomatic uncomplicated gallstones. If determinants for symptom resolution following cholecystectomy are identified, future randomized controlled trials may explore selection strategies yet further to improve management strategies for the treatment of symptomatic uncomplicated gallstones.

## 10. Conclusions

Studies exploring symptomatic outcomes following cholecystectomy mostly include heterogeneous populations with a variety of clinical gallstone presentations. Further, they fail to report a diagnostic workup or treatment for post-cholecystectomy symptoms. This challenges the comparison of symptomatic outcomes between studies. Although biliary pain seems to resolve following cholecystectomy in most patients, dyspeptic symptoms may co-exist with pain and may both resolve or develop following cholecystectomy. Patient satisfaction following cholecystectomy is high, which may reflect a symptom alleviation or, alternatively, a change in symptoms. Persisting complaints following surgery are mostly determined by preoperative functional disorders, atypical pain locations, long duration of symptoms, frequent and long pain episodes, and poor psychological or physical health. Even when the selection of patients for cholecystectomy is restricted to the presence of biliary pain, unacceptably high proportions of patients have persisting pain. Biliary pain is ill-defined and mostly based on consensus statements, but it currently remains the only criterion for cholecystectomy in the presence of symptomatic uncomplicated gallstones. The exploration of patient-reported symptoms only for the prediction of cholecystectomy symptomatic outcome has been exhausted. Future prospective clinical studies should explore the impact of objective determinants for symptomatic gallstones on pain resolution following cholecystectomy in patients with presumed symptomatic uncomplicated gallstones.

## Figures and Tables

**Table 2:** Determinants of postoperative symptoms identified in prospective clinical studies.

Postoperative Outcome	Preoperative Determinant
Symptom resolution	Previous cholecystitis [70]
	Higher age [62]
Pain resolution	No heartburn [78]
	Lower visual analogue scale [78]
	Pain attacks [78]
	Pain awakening at night [77]
	Pain frequency ≤ 1 per month [77]
	Symptom duration ≤ 1 year [77,87]
	No use of pain medication [78]
	No previous abdominal surgery [78]
	Higher Gastrointestinal Quality of Life Index [87]
	Higher age [77,78]
Persisting symptoms	Dyspeptic symptoms [63,70]
	Symptom duration > 6 months [70]
	Gastritis [93]
	Poor self-rated health [70]
	Higher Hopkins Symptom Checklist [81]
	Gastrointestinal Symptom Rating Scale [81]
	Higher trait anxiety [43]
	Higher Psychological Symptom Score [93]
	ASA III–IV [70]
Pain	Dyspepsia [68]
	Functional dyspepsia/irritable bowel syndrome [79]
	Atypical pain locations/lower abdominal pain [74,77]
	Flatulence [74]
	Food intolerance [74]
	Abnormal bowel pattern (diarrhea or constipation) [77]
	Often feeling bloated [77]
	Pain attacks every month [74]
	Psychic vulnerability [83]
Dyspepsia	Dyspepsia [43,68]
	Non-specific symptoms [43]
	Psychotropic medication [43]
	Middle age range (40–69 years) [95]
Unsatisfying/unsuccessful cholecystectomy outcome	Flatulence [59]
	Pain episode duration of days or more [59]
	Symptom duration > 6 months [70]
	Poor self-rated health [70]
	Age > 55 years [59]
	Younger age [62]
Diarrhea	Age < 50 years [92,96]
	Higher body mass index group [96]

**Table 3:** Symptomatic outcome following cholecystectomy in prospective studies.

	Preoperative Proportion (%)	Decline	Incline	Debuting
Pain attacks/biliary colic/biliary pain	42–100%	↓↓↓	–	(0%)
Unspecified/atypical pain	9–100%	↓↓	↑	0–7%
Dyspepsia	11–88%	↓↓	(↑↑↑)	0–6%
Heartburn	25–66%	↓↓	–	1–8%
Regurgitation/reflux	11–72%	↓↓	(↑)	2–14%
Nausea	13–90%	↓↓↓	(↑↑↑)	0–2%
Vomiting	7–54%	↓↓↓	(↑↑↑)	0–1%
Fat or food intolerance	36–80%	↓↓↓	–	2–13%
Indigestion/postprandial heaviness	14–78%	↓↓	–	–
Flatulence	15–88%	↓	–	0–10%
Diarrhea	2–22%	(→)	↑↑↑	14–17%
Bloating/distension	37–82%	↓↓	–	–
Constipation	7–65%	→	–	–

Arrows indicate % change in proportion of patients reporting symptoms before versus after cholecystectomy as reported in most studies. ↓↓↓/↑↑↑ decline/incline of 60% or more; ↓↓/↑↑ decline/incline of 30–60% or more; ↓/↑ decline/incline of 30–60%; → % change less than 30%; – indicates no available studies or incomparable estimates between studies; () indicates estimates from single studies.

## Data Availability

Not applicable.
